# From Bench to Bone: Translational Pathways of Nanomaterial-Based Therapies in Craniomaxillomandibulofacial and Cranial Vault Reconstruction

**DOI:** 10.3390/bioengineering13091014

**Published:** 2026-08-31

**Authors:** Ioannis Chatzistefanou, Sophia Tsokkou, Alexandros C. Liatsos, Kyriaki Papadopoulou, Georgia-Alexandra Spyropoulou, Vasileios Petsinis, Theodora Papamitsou

**Affiliations:** 1 University General Hospital “Attikon”, National and Kapodistrian University of Athens, 12462 Athens, Greece; 2 Laboratory of Histology-Embryology, School of Medicine, Faculty of Health Sciences, Aristotle University of Thessaloniki, 54124 Thessaloniki, Greece; 3 Department of Neurosurgery, AHEPA University Hospital, Aristotle University of Thessaloniki, 54636 Thessaloniki, Greece; 4 Clinic of Plastic and Reconstructive Surgery, Papageorgiou General Hospital, Aristotle University of Thessaloniki, 56429 Thessaloniki, Greece

**Keywords:** nanomaterials, craniomaxillofacial reconstruction, cranial vault, bone regeneration, hydroxyapatite, mesoporous silica nanoparticles, 3D printing, osteoimmunomodulation, angiogenesis

## Abstract

Craniomaxillofacial and cranial vault reconstruction remain limited by donor-site morbidity, infection, inadequate vascularization, incomplete osseointegration, and poor adaptation of conventional grafts to complex patient-specific anatomy. Nanomaterial-based therapies offer a translationally attractive strategy. Nanoscale hydroxyapatite, calcium phosphates, mesoporous silica, metallic nanoparticles, bioactive glasses, polymeric carriers, injectable nanocomposite hydrogels, extracellular vesicle-inspired systems, and nanostructured coatings can act on several targets at once, including osteogenesis, angiogenesis, immune response, antimicrobial activity, mechanical reinforcement, and controlled release. This review synthesizes recent evidence on nanomaterial-enabled bone regeneration for craniomaxillofacial and cranial vault reconstruction, placing emphasis on the pathway from in vitro mechanisms to animal models, additive manufacturing, patient-specific implants, and early clinical translation. 3D-printed calcium phosphate or hydroxyapatite-based patient-specific scaffolds are currently supported by the strongest near-clinical evidence, whilst immunomodulatory, bioprinted, injectable, and drug-delivering nanocomposites remain largely preclinical. Growth-factor-independent strategies based on ionic signaling, dipyridamole-enhanced adenosine signaling, nanosilicate-mediated osteogenesis, Wnt/beta-catenin mechanotransduction, and nanoparticle-mediated osteoimmunomodulation may reduce reliance on recombinant bone morphogenetic proteins. However, large-animal validation, long-term biodistribution, nanotoxicology, sterilization, manufacturing reproducibility, and regulatory classification remain major barriers. Future clinical translation will require rational scaffold design, anatomical indication-specific testing, antimicrobial integration, and harmonized regulatory evidence packages.

## 1. Introduction

Reconstruction of craniomaxillofacial and cranial vault defects occupies a demanding intersection between function, esthetics, infection control, skeletal biology, and patient-specific geometry. After trauma, tumor resection, congenital deformity correction, decompressive craniectomy, craniosynostosis surgery, alveolar cleft repair, orthognathic revision, implant-related failure, or osteonecrosis, various defects may arise. Unlike many appendicular skeletal defects, craniofacial reconstruction must restore contour, protect critical neurovascular and ocular structures, preserve occlusion or airway function where relevant, and integrate with thin, irregular, often poorly vascularized bone margins. Regarding pediatric reconstructions, cranial growth, suture patency, future symmetry, and avoidance of rigid non-resorbable constructs that may interfere with development all constitute additional concerns that need to be considered.

Autologous bone remains the clinical benchmark, inasmuch that it provides osteogenic cells, osteoinductive factors, and an osteoconductive matrix. Nevertheless, donor-site morbidity, limited volume, unpredictable resorption, contour mismatch, prolonged operative time, and morbidity at secondary harvest sites limit autografting. Allografts and xenografts, on the other hand, reduce donor-site morbidity, but they still introduce concerns when it comes to biological activity, immune reaction, disease transmission, variable remodeling, and incomplete integration. Conventional synthetic material, such as titanium, polyetheretherketone (PEEK), polymethylmethacrylate (PMMA), and bulk calcium phosphate ceramics, provide structural replacement, but may lack active biological signaling, antimicrobial protection, or full osteointegration. Current clinical consensus in craniomaxillofacial bone regeneration continues to identify vascularization, biologically active integration, controlled resorption, infection prevention, and reliable clinical evidence as central unresolved challenges [1].

Nanomaterials address several of these problems because bone itself is a hierarchical nanocomposite. The native mineralized extracellular matrix contains nanoscale apatite crystals embedded within collagen fibrils, producing a surface chemistry and topography that regulate protein adsorption, cell adhesion, osteoblast differentiation, osteoclast activity, immune signaling, and mineral deposition. Engineered nanomaterials can therefore be used not merely as miniature fillers but as functional biological interfaces. Nanoscale hydroxyapatite, calcium phosphate, bioactive glass, silica, iron oxide, magnesium phosphate, gold, silver, copper, zinc oxide, titanium dioxide, graphene derivatives, polymeric nanoparticles, extracellular vesicle-inspired systems, and nanostructured implant surfaces have all been explored for skeletal and craniofacial regeneration [2,3,4,5,6,7].

The translational promise is particularly strong in craniomaxillofacial surgery because patient-specific anatomy can be captured by computed tomography or magnetic resonance imaging and converted into computer-aided design/computer-aided manufacturing workflows. Additive manufacturing enables personalized implants with controlled macrogeometry, pore architecture, surface topography, and local release systems. 3D-printed titanium, PEEK, hydroxyapatite, alpha-tricalcium phosphate, beta-tricalcium phosphate, and composite scaffolds have already entered preclinical and selected clinical craniofacial applications [8,9,10,11]. The next translational step is to integrate nanoscale biological activity into these patient-specific platforms without compromising safety, manufacturability, sterilization, or regulatory viability.

This review synthesizes recent evidence on nanomaterial-based therapies for craniomaxillofacial and cranial vault reconstruction. Rather than treating nanomaterials as a single class, it organizes them by function: osteoconductive/osteoinductive mineral systems, nanoparticle-reinforced hydrogels, immunomodulatory platforms, angiogenic delivery systems, antimicrobial coatings, 3D-printed and bioprinted constructs, and regulatory-ready patient-specific implants. Defining the translational pathway from bench mechanisms to clinically relevant reconstruction constitutes the central aim of this study.

## 2. Review Scope

The review focuses on critically synthesizing contemporary evidence on nanomaterial-enabled strategies for craniomaxillofacial and cranial vault reconstruction, with specific emphasis on their translational progression from fundamental mechanisms to clinically relevant applications. The review examines diverse nanomaterial platforms, ranging from mineral-based systems and bioactive glasses to mesoporous carriers, metallic nanoparticles, injectable nanocomposites, and 3D-printed patient-specific constructs. For each platform, various parameters are being considered, among them the modulation of osteogenesis, angiogenesis, immune responses, antimicrobial activity, and mechanical performance. By mapping findings across in vitro studies, small- and large-animal cranial models, and early human case applications, the review analyzes the current state of translational readiness while identifying material classes with near-clinical potential, underscoring persistent barriers related to safety, manufacturing, and regulatory adoption. In addition, the review uses the term craniomaxillomandibulofacial deliberately, covering four biologically distinct territories—the cranial vault, the orbito-midfacial skeleton, the maxilla and dentoalveolar complex, and the mandible—and treats the maxilla and mandible separately (Section 7.2 and Section 7.3) since they differ in developmental template, architecture, vascular supply, microbial exposure, and mechanical loading.

Existing reviews address adjacent ground but either focus on molecular mechanisms without anatomical specificity [2,3,4,5], materials or manufacturing [6,8,9,12,13], regulatory science in general terms [10], or broad craniofacial scaffold overviews [14,15,16]. The present work differs by organizing evidence by anatomical indication rather than material class, by placing every platform on an explicit translational-readiness ladder from in vitro proof of mechanism to regulatory adoption, and by integrating nanotoxicology, sterilization, manufacturing reproducibility, and EU MDR/FDA classification into the material-by-material discussion rather than appending them as a closing caveat.

## 3. Clinical Problem: Why Craniofacial Reconstruction Needs Active Nanomaterial Platforms

### 3.1. Anatomical and Biological Constraints

From a purely biological standpoint, craniomaxillofacial reconstruction differs from long-bone repair. Craniofacial bones originate from neural crest and mesodermal lineages, contain variable cortical–cancellous composition, and exist in close proximity to sinus, oral, nasal, orbital, and intracranial compartments. Many defects are thin, curved, irregular, or contaminated. Bone margins may be compromised by prior radiation, infection, tumor surgery, osteomyelitis, or repeated hardware failure. The cranial vault is relatively non-load-bearing but must protect intracranial contents and preserve contour. The mandible is load-bearing and exposed to occlusal forces, oral bacteria, and soft tissue tension. The orbit and midface demand millimeter-level shape accuracy to avoid diplopia, enophthalmos, asymmetry, and functional impairment.

These anatomical constraints mean that an ideal reconstructive material must do more than fill space.

It should be biocompatible, shape-stable, osteoconductive, and preferably osteoinductive.

Moreover, it should also permit vascularization, resist infection, and remain radiologically assessable. The reconstructive material should also be surgically workable, mechanically matched to the target site, and manufacturable under regulatory standards. Foundational craniofacial scaffold criteria also emphasize interconnected porosity, matched degradation, vascular ingrowth, and site-specific resorption, with pediatric craniofacial applications requiring particular attention to growth compatibility and avoidance of persistent rigid barriers [15]. No current platform fully satisfies these criteria across all craniofacial indications.

### 3.2. Limitations of Current Standards

Consensus-based clinical evidence supports autologous bone as the gold standard for craniomaxillofacial bone regeneration, but its limitations are substantial: donor-site morbidity, volume restriction, resorption, and variable contour stability [1,14,15,17]. Deproteinized bovine bone mineral and other xenografts provide osteoconduction and volume stability but lack living osteogenic components [1]. Allografts reduce donor-site morbidity but raise concerns regarding biological activity, immune response, and disease transmission [1]. Recombinant human bone morphogenetic protein-2 has regulatory approval for selected oral and maxillofacial indications, but supraphysiological dosing can be associated with edema, ectopic bone formation, osteoclast-mediated resorption, and immunogenicity [1,15,16].

Conventional alloplastic reconstruction materials also have limitations. Titanium provides strength and imaging compatibility but is biologically inert unless surface-modified, and may be complicated by exposure or infection [18,19]. PEEK offers excellent shape stability and mechanical performance but lacks osteoconduction, and may permit bacterial adhesion. PMMA is inexpensive and shapeable but does not remodel or integrate biologically. Hydroxyapatite and calcium phosphate ceramics are more bone-like and osteoconductive but can be brittle, initially weak, and limited in load-bearing applications [8,20,21].

### 3.3. The Translational Opportunity

Nanomaterial platforms can be designed to target these failure modes. Nanostructured minerals provide osteoconductive chemistry and protein-binding surfaces. Mesoporous nanoparticles can deliver aspirin, VEGF, BMPs, antibiotics, nucleic acids, or small molecules with controlled release. Metallic nanoparticles can modulate macrophage phenotype or provide antibacterial activity. Bioactive glass and multi-ionic ceramics release calcium, phosphate, silicon, magnesium, strontium, copper, iron, or zinc ions that activate osteogenic and angiogenic signaling. Nanotopographic coatings can improve osseointegration or physically deter bacterial adhesion. When combined with 3D printing, these properties can be spatially organized within patient-specific constructs.

Three platforms merit immediate translational investment, inasmuch that in each case the remaining evidence gap is definable and finite rather than open-ended. The first is the 3D-printed calcium phosphate or hydroxyapatite patient-specific implant for non-load-bearing cranial vault and orbito-midfacial contour defects. The missing element here is not the mechanism but prospective comparative clinical data against titanium, PEEK, and autograft, with volumetric and imaging endpoints at 24 months or beyond; a realistic horizon could be three to five years. The second is the dipyridamole-coated resorbable beta-tricalcium phosphate scaffold, which already carries large-animal cranial and pediatric model data together with a long systemic drug-use precedent, and which now requires a first-in-human study with an explicit dose-per-surface-area justification; a realistic horizon could be five years. The third is the antimicrobial and osseointegrative nanotopographic surface on titanium or PEEK, where low-release or no-release surface architecture avoids the systemic exposure problem inherent to dissolving metallic nanoparticles, and where infected-defect large-animal models are the missing step; a realistic horizon could be three to five years as well.

## 4. Nanomaterial Classes and Mechanisms Relevant to Craniofacial Bone Regeneration

### 4.1. Hydroxyapatite and Calcium Phosphate Nanomaterials

Hydroxyapatite is the most intuitive mineral platform because it resembles native bone mineral. Nanoscale hydroxyapatite increases surface area, protein adsorption, and osteoblast interaction compared with bulk materials. Hydroxyapatite-based patient-specific implants and uncalcined/unsintered hydroxyapatite composite sheets provide osteoconductive surfaces for bone deposition, and may support gradual replacement or integration [20,21] (Table 1).

In a rat mandibular defect model, uncalcined/unsintered hydroxyapatite combined with poly-L-lactide-co-glycolide showed osteoconductivity comparable to the established uncalcined/unsintered hydroxyapatite/poly-L-lactide comparator, with accelerated early bone formation and an estimated shorter resorption time [21]. This is relevant for maxillofacial reconstruction because bioresorbable fixation or barrier sheets could reduce the need for secondary removal and minimize late foreign-body complications. However, rodent mandibular models do not replicate human bite forces, defect dimensions, or long-term functional loading (Table 1).

Three-dimensional-printed hydroxyapatite bioceramic patient-specific implants represent a more clinically advanced translation. A scoping review with two human craniomaxillofacial clinical cases reported satisfactory esthetic and functional outcomes, stability, absence of infection, and radiological signs of osteogenesis at short follow-up [20]. These implants are particularly attractive for load-sharing or non-load-bearing defects such as orbital rim, supraorbital, contour, or selected mandibular angle reconstructions. Their limitations include brittleness, lower initial mechanical strength compared with titanium or PEEK, short follow-up data, and uncertain suitability for load-bearing or dental implant-bearing regions (Table 1).

Beta-tricalcium phosphate is another major calcium phosphate platform. It is resorbable, osteoconductive, printable, and can be functionalized with pharmacological coatings such as dipyridamole. Translational studies reviewed by Slavin et al. describe 3D-printed beta-tricalcium phosphate scaffolds coated with dipyridamole, with optimized pore size, cap geometry, and evidence across rabbit, sheep, and minipig cranial models [11]. The finding that dipyridamole-coated scaffolds were non-inferior to autografts in a skeletally immature craniofacial model is especially important because pediatric cranial reconstruction remains constrained by growth, suture preservation, and autograft morbidity [11] (Table 1).

Recent maxillofacial studies extend calcium phosphate translation into thermoplastic and electrospun nanocomposites. A beta-TCP/PDLLA nanobiomaterial demonstrated intraoperative cuttability and thermal reshaping in a rat mandibular critical-size defect model, with early osteocalcin expression and new bone within scaffold pores [34]. A cotton-like electrospun beta-TCP/PLLA/PGA material achieved comparable osteoconductive performance to pure beta-TCP in rat mandibular defects while using less implanted material and showing early periosteal rim closure, RUNX2 expression, LepR-positive cell recruitment, and periostin activity [35]. These platforms are clinically relevant because irregular mandibular and alveolar defects often require graft materials that can be packed, trimmed, or reshaped without losing osteoconductive function (Table 1).

Polymer–calcium phosphate nanocomposites also provide direct calvarial evidence. 3D-printed PLA scaffolds filled with gelatin–nanohydroxyapatite and coated with platelet-rich plasma produced greater rat calvarial defect fill than PLA alone, reaching 69.8% and 83.7% of new bone area at 8 and 12 weeks in the PRP-containing group [32]. A PLA/hardystonite–graphene oxide scaffold loaded with simvastatin through a microporous pectin-quaternized chitosan polyelectrolyte produced sustained release and near-complete rat calvarial healing at 8 weeks [33]. Electrospun PCL–nanohydroxyapatite scaffolds have also shown increased osteopontin, osteocalcin, ALP activity, mineral deposition, and in vivo bone continuity in a rabbit defect model, supporting nanohydroxyapatite as a recurring osteogenic additive across polymer families [22] (Table 1).

Overall, hydroxyapatite and calcium phosphate nanomaterials are among the most realistic near-clinical platforms for craniomaxillofacial and cranial vault reconstruction, particularly for non-load-bearing contour defects, cranial vault defects, orbital rim or supraorbital reconstruction, and selected mandibular or alveolar adjunctive indications. Their translational strength lies in their chemical similarity to native bone mineral, osteoconductivity, compatibility with 3D printing, and existing clinical familiarity. The main barriers are brittleness, limited load-bearing capacity, variable resorption, short follow-up in human patient-specific implant studies, and insufficient comparative data against titanium, PEEK, autograft, or established calcium phosphate substitutes. The next required evidence consists of prospective clinical cohorts and controlled comparative studies assessing implant stability, osseointegration, infection, revision surgery, imaging outcomes, patient-reported esthetic results, and long-term performance in anatomically defined craniofacial indications. The likely clinical timeline is near-term for acellular patient-specific calcium phosphate or hydroxyapatite implants, and intermediate-term for pharmacologically enhanced or polymer–calcium phosphate nanocomposites (Table 1).

Critical appraisal: For the aforementioned class, evidence encompasses early human experience in small patient-specific implant series [20], supported by rodent [21,22] and large-animal cranial data [11,23], yet the limitations remain mechanical and kinetic. To non-load-bearing sites is confined brittle sintered hydroxyapatite, bone deposition in compromised beds can be outpaced by beta-tricalcium phosphate resorption, and resorption exhibits different features between calvarial and alveolar sites. Since no published series includes a comparator, the unresolved question is whether these implants outperform titanium or PEEK, which would require a prospective comparative or randomized cranial vault trial with implant survival, infection, revision, and volumetric contour retention at 24 months as endpoints (Table 1).

### 4.2. Bioactive Glass, Silicate, and Multi-Ionic Nanoparticles

Bioactive glass and silicate-based nanomaterials release biologically active ions that stimulate osteogenesis and angiogenesis. Silicon, calcium, magnesium, strontium, copper, cobalt, and related ions can activate MAPK/ERK, Wnt/beta-catenin, PI3K/Akt, HIF-1 alpha/VEGF, RUNX2, alkaline phosphatase, osteocalcin, and collagen pathways [2,3,36,37,38,39]. This makes multi-ionic systems attractive as growth-factor-sparing platforms (Table 1).

Merwinite nanoparticles, composed of calcium, magnesium, and silicate phases, have been incorporated into 3D-printed hyaluronic acid–fucoidan scaffolds with recombinant VEGF for craniofacial osteoangiogenesis [28]. The combined platform improved compressive properties, apatite deposition, rat bone marrow mesenchymal stromal cell osteogenic markers, and chorioallantoic membrane vascularization. However, it remains below the level of cranial defect animal validation, and the inclusion of recombinant VEGF introduces combination-product complexity (Table 1).

Magnesium phosphate-based bioinks provide another growth-factor-independent strategy. An extracellular matrix-like peptide hydrogel combined with amorphous magnesium phosphate supported dental pulp stem cell viability after printing, increased RUNX2, COL1A1, osteopontin, alkaline phosphatase activity, and mineralization, and improved bone volume fraction in a rat calvarial defect model [27]. The system is attractive because magnesium ion release can stimulate osteogenesis without recombinant BMP-2. Its limitations include incomplete defect closure, absence of large-animal validation, and regulatory complexity if cell-laden constructs are used clinically (Table 1).

Nanosilicate systems provide a particularly important bridge between bioactive glass chemistry and printable hydrogels. Laponite nanosilicate incorporated into gelatin–alginate bioink improved printability, doubled the compressive modulus compared with gelatin–alginate alone, supported rBMSC viability, and induced osteogenic differentiation without osteogenic medium; in rat calvarial defects, the 2% nanosilicate cell-laden bioprinted scaffold achieved the highest BV/TV at 12 weeks and produced cortical-like lamellar bridging [40]. Organic–inorganic poly(citrate–siloxane) nanoparticles further suggest that siloxane-containing nanoparticles can activate osteogenesis through defined materiobiological interactions, including CCN3 anchoring and downstream Wnt10b/beta-catenin activation [41] (Table 1).

Magnesium, strontium, and copper would each require appraisal of their therapeutic window, since the translational variable is released dose over time rather than ion identity [42]. Magnesium stimulates osteoblast adhesion and angiogenesis through integrin and HIF/VEGF signaling and carries a strong device safety precedent [2,3,27,43,44,45,46], but its release is often too rapid to sustain across craniofacial healing and can cause transient alkalinization or, in metallic form, hydrogen evolution, so it is best delivered from a slowly dissolving ceramic or bioink phase. Strontium acts on both arms of remodeling via calcium-sensing receptor/Wnt signaling and the OPG/RANKL axis and is radiopaque [2,3,36,47]; however, excessive substitution yields hypomineralized bone, and systemic strontium ranelate safety history obliges explicit documentation of local and systemic exposure, favoring low substitution in a slowly resorbing carrier. Copper offers the strongest dual rationale, driving HIF-1α/VEGF-mediated angiogenesis at low micromolar levels while remaining antibacterial at higher ones [30,31,36,47], yet the same Fenton-type redox activity narrows its selectivity index against osteoblasts and endothelial cells, making it defensible only as a low-dose, slowly released, or surface-confined component. In an overall analysis, the best safety-to-benefit ratio for bulk incorporation is offered by magnesium, the most useful remodeling balance at low substitution by strontium, and the greatest payoff at the greatest formulation risk by copper. Nonetheless, for all three elements reporting of released ion concentration over time within the defect volume is required, rather than total ion content (Table 1).

Ultimately, bioactive glass, silicate, magnesium, and multi-ionic nanomaterials are translationally promising for growth-factor-sparing craniofacial bone regeneration, especially where osteogenesis and angiogenesis need to be stimulated without high-dose recombinant BMPs or VEGF. Their major advantage is the ability to activate endogenous osteogenic, angiogenic, and mechanotransductive pathways through controlled ion release and scaffold chemistry. However, most platforms remain preclinical. Their translation is limited by incomplete defect closure in some models, by uncertain ion-release kinetics at human scale, by regulatory complexity when cells or recombinant growth factors are added, and by limited large-animal craniofacial validation. The next required evidence is a staged program of cranial or mandibular large-animal studies comparing ion-release scaffolds against calcium phosphate controls. These studies should quantify bone volume, vascularization, degradation, local ion concentrations, systemic exposure, and mechanical performance. The likely clinical timeline is intermediate-term for acellular ion-releasing scaffolds and long-term for cell-laden or recombinant-factor-containing bioinks (Table 1).

Critical appraisal: Ion-releasing and silicate systems reach only rodent calvarial validation [27,40], with recurring limitations regarding incomplete defect closure, release kinetics characterized in static immersion rather than perfused defects, narrow therapeutic windows overlapping cytotoxic ranges, and loss of regulatory simplicity whenever ions are combined with recombinant VEGF or cells. Whether ionic stimulation alone can bridge a critical-size defect at human scale remains unresolved, and the next required study is proposed to be a large-animal cranial or mandibular comparison of an acellular ion-release scaffold against a calcium phosphate control, reporting released ion concentrations over time alongside bone volume, vessel density, and mechanical outcome (Table 1).

### 4.3. Mesoporous Silica Nanoparticles

Mesoporous silica nanoparticles are versatile because they combine high surface area, tunable pore size, cargo loading, controlled release, and silicon-mediated osteogenic signaling. In cranial defect models, mesoporous silica has been used as a carrier for aspirin and as a scaffold for gold nanoparticle decoration [24,25]. Aspirin-loaded mesoporous silica within chitosan hydrogel produced sustained drug release, macrophage polarization toward an anti-inflammatory/pro-reparative phenotype, upregulation of TGF-beta/Smad signaling, and substantial bone regeneration in rat critical-size cranial defects [24].

Gold-loaded mesoporous silica in chitosan scaffolds similarly promoted macrophage M2 polarization, secretion of BMP-2, TGF-beta1, and VEGF, direct osteogenic differentiation of preosteoblastic cells, a reduced RANKL/OPG ratio, and accelerated bone formation in rat cranial defects [25]. Together, these studies support the concept that mesoporous silica is not simply a passive drug carrier but a programmable osteoimmunomodulatory platform (Table 1).

The main translational concerns are long-term silica biodistribution, degradation kinetics, local and systemic nanoparticle exposure, size-dependent cytotoxicity, manufacturing reproducibility, and lack of large-animal or human craniofacial evidence (Table 1).

Overall, mesoporous silica nanoparticles are promising for immunomodulatory and drug-delivering cranial defect applications, particularly where controlled release and macrophage-mediated osteogenesis are desired. Strong mechanistic evidence supports their capacity to function as programmable osteoimmunomodulatory systems rather than as passive carriers. However, the current evidence does not yet justify clinical translation because long-term silica biodistribution, degradation, dose-dependent toxicity, local and systemic nanoparticle persistence, and large-animal craniofacial performance remain insufficiently defined. The next required step is a large-animal calvarial or mandibular defect study including quantitative biodistribution, degradation analysis, immune profiling, local and systemic toxicity, and direct comparison against calcium phosphate, autografting, or clinically established scaffold controls. The likely clinical timeline is intermediate- to long-term, depending on whether safety and clearance can be demonstrated convincingly (Table 1).

**Figure 1:**
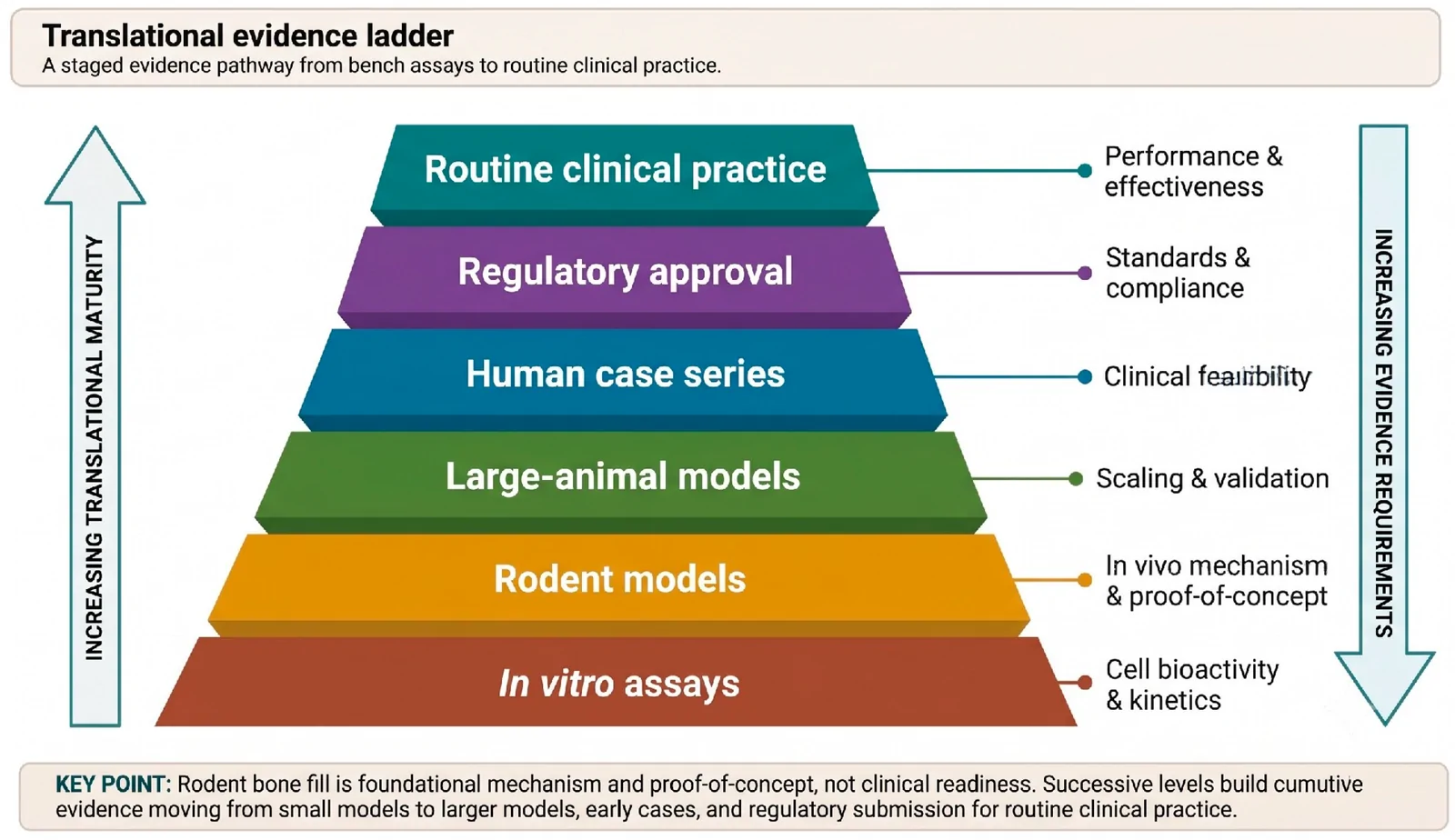
Translational evidence ladder.

Critical appraisal: Mesoporous silica reaches rodent cranial defect validation with strong mechanistic support for immunomodulation [24,25], but silica degradation has not been quantified over a clinically relevant period, biodistribution and organ accumulation remain unreported, the cargo rather than the carrier may drive much of the effect, and loading reproducibility and sterilization stability have not been addressed at all. What fraction of the particle mass remains in the defect at complete healing is therefore unknown, and the next required study is a labeled biodistribution and clearance experiment in a large-animal cranial defect with terminal organ analysis and a carrier-only control arm (Table 1 and Table 2), (Figure 1).

### 4.4. Metallic and Metal Oxide Nanoparticles

Metallic nanoparticles can provide osteogenic, angiogenic, magnetic, antimicrobial, and immunomodulatory functions. Gold nanoparticles have been associated with macrophage modulation and osteogenic signaling, although their biological effects are dose-, size-, and surface chemistry-dependent [25]. Iron oxide nanoparticles can provide iron-mediated angiogenic signaling, mechanical reinforcement, and magnetic imaging potential. A functional iron oxide nanoparticle-crosslinked methacrylated gelatin hydrogel improved stiffness, osteogenesis, angiogenesis, and bone regeneration in a rat cranial defect model, while also offering MRI-trackable scaffold potential [26] (Table 1).

Silver, copper, zinc oxide, and titanium dioxide nanoparticles are mainly discussed in antimicrobial applications. Silver nanoparticles provide broad antibacterial and antibiofilm activity but have narrow therapeutic windows and systemic accumulation concerns [30]. Copper nanoparticles are particularly interesting because copper ions may stimulate VEGF-mediated angiogenesis at sub-antimicrobial concentrations while exerting bactericidal effects at higher concentrations [30]. Zinc oxide and titanium dioxide provide ROS-mediated antimicrobial effects, but photocatalytic mechanisms may be less useful in deep implanted craniofacial sites unless activation can be achieved intraoperatively or by alternative energy delivery (Table 1).

Metallic nanomaterials are therefore powerful but translationally delicate. Their clinical application will require precise release control, coating stability, dose limits, chronic toxicity assessment, and proof that antimicrobial efficacy does not compromise osteoblasts, endothelial cells, or resident immune cells.

Graphene derivatives and carbon nanotubes illustrate both the promise and caution of carbon-based nanomaterials. Reviews of craniofacial and dental tissue engineering report graphene-associated osteogenic differentiation through substrate stiffness and BMP-2-related signaling, as well as carbon nanotube and titanium dioxide nanoscaffold data in skull models [14,49]. Nevertheless, non-biodegradability, inflammatory potential, pulmonary exposure during manufacturing, and an uncertain long-term fate currently limit their direct clinical use in implanted craniofacial scaffolds [49] (Table 1).

Ultimately, metallic and metal oxide nanoparticles are most promising as adjunctive technologies rather than stand-alone reconstructive scaffolds. Iron oxide nanoparticles may have an intermediate-term translational pathway as mechanically reinforcing, angiogenic, and potentially image-trackable components of hydrogels or coatings, whereas silver, copper, zinc oxide, and titanium dioxide nanoparticles are more relevant for antimicrobial and antibiofilm surface modification of titanium.

The major limitation of PEEK and calcium phosphate implants is their narrow safety window between antimicrobial or angiogenic activity and mammalian-cell toxicity. Further concerns include chronic accumulation, oxidative stress, inflammatory activation, and release control. The next required evidence includes dose-ranging studies, long-term local and systemic toxicity testing, infected craniofacial defect models, and comparison between release-based antimicrobial systems and low-release nanotopographic surfaces.

Critical appraisal: Rodent cranial validation for iron oxide hydrogels [26], a clinical precedent for silver and copper surfaces outside craniofacial bone [30], and in vitro and early rodent work for graphene and carbon nanotubes [14,49] all emerge as evidence for this category, with correspondingly specific limitations. Among the latter are a narrow selectivity index between antimicrobial and cytotoxic concentrations, an indefinite residence time for non-biodegradable gold, graphene, and carbon nanotubes, absent long-term iron handling data, and photocatalytic mechanisms that are impractical in deep implant sites. Whether any freely releasing metallic nanoparticle system can sustain antimicrobial efficacy at cell-tolerated concentrations over weeks remains unresolved. A proposed next step is a dose-ranging experiment in an infected craniofacial defect model comparing release-based against non-releasing nanotopographic surfaces, and measuring bacterial burden, bone formation, and local cytotoxicity in the same animals (Table 1 and Table 2).

### 4.5. Polymeric Nanoparticles, Lipid Nanoparticles, and Extracellular Vesicle-Inspired Systems

Polymeric nanoparticles such as PLGA, PEG, chitosan, dendrimers, and related carriers are used to deliver growth factors, anti-inflammatory drugs, antibiotics, nucleic acids, and osteogenic molecules [2,3,5]. PLGA is especially attractive because it has a clinical precedent and predictable degradation products. VEGF-loaded PLGA microspheres integrated into 3D-printed gelatin/alginate/beta-TCP scaffolds produced biphasic VEGF release, increased HUVEC proliferation, and enhanced alkaline phosphatase activity in vitro [29]. This early platform illustrates the logic of local angiogenic delivery but requires in vivo validation (Table 1).

Lipid nanoparticles and extracellular vesicle-inspired systems are emerging as delivery vehicles for mRNA, siRNA, miRNA, and paracrine signaling. Their relevance to craniofacial reconstruction lies in precision modulation of osteogenesis, angiogenesis, and macrophage phenotype. However, compared with mineral or ceramic scaffolds, they remain less mature for implantable cranial reconstruction and face substantial regulatory, manufacturing, and biodistribution questions [2,5] (Table 1).

Injectable nanomaterial-integrated hydrogels are a rapidly expanding subcategory because they can conform to irregular craniofacial cavities and deliver mechanical, osteogenic, immunomodulatory, angiogenic, and antimicrobial cues in situ. Polymeric nanocomposite hydrogel reviews describe natural, synthetic, and hybrid matrices reinforced with nanohydroxyapatite, carbon nanomaterials, metallic nanoparticles, and nanoclays, including 3D-printed cryogel or hydrogel scaffolds for craniofacial cleft-type defects [50]. A craniofacial-specific review of nanomaterial-integrated injectable hydrogels maps ceramic, silicon-based, metal, carbon, organic, drug-loaded, peptide, and exosome-containing systems to macrophage polarization, Wnt/beta-catenin, PI3K/Akt, MAPK, BMP/Smad, HIF-1 alpha/VEGF, antibacterial, and stress-relaxation mechanisms [31]. These platforms are attractive for minimally invasive or geometrically complex sites, but evidence remains dominated by rodent and rabbit models with limited long-term biodistribution data (Table 1).

Overall, polymeric nanoparticles and injectable nanocomposite hydrogels are promising for irregular craniofacial cavities, alveolar or sinus augmentation, local angiogenic or antimicrobial delivery, and minimally invasive adjunctive reconstruction. PLGA-based delivery systems have the strongest translational logic within this group because of their established biomedical precedent and degradability. However, lipid nanoparticles, extracellular vesicle-inspired systems, and nucleic acid-based delivery remain speculative for implantable craniofacial reconstruction because their biodistribution, dosing, manufacturing reproducibility, storage, release kinetics, and regulatory classification are not yet sufficiently resolved. Injectable nanocomposite hydrogels also require better control over in situ gelation, mechanical stability, degradation, and retention in defects exposed to saliva, sinus cavities, soft tissue motion, or occlusal forces. The next required evidence is in vivo validation in anatomically relevant craniofacial models, with attention to retention, integration, vascularization, biodistribution, immune response, and comparison against standard grafting materials. The likely clinical timeline is intermediate-term for acellular PLGA- or nanoceramic-reinforced injectable systems, and long-term for extracellular-vesicle, lipid-nanoparticle, or nucleic-acid-based constructs (Table 1).

Critical appraisal: Polymeric carriers have reached rodent and rabbit preclinical work while lipid nanoparticle and extracellular vesicle systems remain largely in in vitro proof-of-concept stages [29,31], limited by acidic autocatalytic degradation of PLGA and PLLA within enclosed craniofacial defects, the semi-permanent behavior of slowly degrading polycaprolactone, the combination-product burden imposed by biologic cargoes such as VEGF or BMP-2, and the absence of potency assays, source-cell characterization, and batch definition for vesicle systems. Whether vesicle-based signaling can be standardized into a product rather than a laboratory phenomenon constitutes till now an open question. A comparative degradation and inflammation experiment contrasting polymer-only with polymer-ceramic composite scaffolds, measuring local pH, inflammatory scoring, and mechanical retention across the full degradation period, is recommended as the next required study (Table 1).

## 5. Biological Mechanisms: From Passive Scaffolds to Active Regenerative Microenvironments

### 5.1. Osteoconduction and Osteoinduction

Nanomaterial scaffolds promote osteoconduction by presenting bone-like mineral chemistry, nanoscale roughness, and high surface energy that favor protein adsorption, integrin engagement, osteoblast adhesion, and mineral nucleation. Hydroxyapatite and calcium phosphate nanoparticles are central to this process. Osteoinduction can be achieved by exogenous biological factors such as BMP-2, but recent nanomaterial strategies increasingly use intrinsic ionic cues, pharmacological coatings, or immune-mediated paracrine signaling.

Growth-factor-independent osteogenesis is a major translational design axis. Magnesium phosphate bioinks stimulate RUNX2, COL1A1, and osteopontin without BMP-2 [27]. Dipyridamole-coated beta-TCP scaffolds act through adenosine A2A receptor signaling and osteoclast modulation, offering a pharmacological enhancement strategy with prior human drug-use precedent [11]. Multi-ionic ceramics release calcium, silicon, magnesium, strontium, copper, or iron ions to activate osteogenic pathways while avoiding high-dose recombinant growth factors [2,3,28].

The added mechanistic literature suggests convergence around Wnt/beta-catenin, YAP/TAZ mechanotransduction, and biomaterial–protein interactions. Nanoparticulate mineralized collagen scaffolds show stiffness-dependent activation of integrin/FAK, YAP/TAZ, Wnt3A/4/11, and active beta-catenin in primary human MSCs, separating mechanical signaling from calcium/phosphate-driven BMPR/Smad effects [51]. Poly(citrate–siloxane) nanoparticles provide an even more molecularly resolved example, where inorganic silanol and organic carboxyl groups bind CCN3, expose Wnt10b-interacting domains, and activate Wnt10b/beta-catenin-mediated osteogenesis [41]. Together with nanosilicate bioinks that induce growth-factor-free calvarial healing [40], these studies support a shift from viewing nanomaterials as passive osteoconductive fillers toward understanding them as instructive regulators of cell fate.

### 5.2. Osteoangiogenic Coupling

Vascularization is a rate-limiting step for large craniofacial and cranial vault defects. Bone formation requires oxygen, nutrient supply, immune cell trafficking, progenitor recruitment, and waste removal. Inadequate vascularization leads to central necrosis, fibrous encapsulation, infection, or scaffold failure. This is particularly relevant in large 3D-printed constructs and irradiated or scarred craniofacial beds.

Nanomaterials can support vascularization through VEGF delivery, hypoxia-mimetic ion release, iron-mediated HIF-1 alpha signaling, copper-stimulated endothelial activation, magnesium and silicon ion effects, and scaffold pore architecture. VEGF-loaded PLGA microspheres demonstrate the principle of controlled angiogenic release [29]. Merwinite–VEGF scaffolds combine multi-ionic and recombinant growth-factor strategies [28]. Iron oxide nanoparticle hydrogels activate HIF-1 alpha/VEGF and PI3K/Akt signaling [26]. These approaches must be balanced against risks of off-target vascularization, burst release, inflammatory angiogenesis, and regulatory classification as drug-device or biologic-device combination products.

This balance is critical for various distinct reasons, each with its own failure mode. First, vessel quality depends on release kinetics rather than on total dose: a burst of VEGF produces numerous immature, hyperpermeable vessels that leak, regress, and never mature into the perfused network that osteogenesis requires, whereas sustained low-level release produces fewer but functional vessels. Second, angiogenesis and osteogenesis must be temporally coupled, since H-type vessel formation precedes and organizes mineralization; an angiogenic stimulus exhausted within the first two weeks has been spent before the osteogenic phase can exploit it. Third, recognized risks, including edema, disorganized ectopic vascular growth, hemangioma-like malformation at supraphysiological doses in animal studies, and inflammatory rather than reparative angiogenesis, are carried by unlocalized or excessive angiogenic signaling. More specifically, in craniofacial reconstruction after tumor resection, sustained pro-angiogenic signaling within a bed that may harbor residual disease arises as a serious concern, which needs to be addressed explicitly. Fourth, direct regulatory consequences stem from the choice: a medical device can remain a scaffold whose angiogenic effect derives from ionic or topographic cues, whilst another delivering a recombinant growth factor could become a drug-device or biologic-device combination product, being characterized by a substantially longer approval pathway. On this basis, matching release kinetics to the healing timeline, confining the stimulus spatially to the defect, and preferring the weakest stimulus that achieves functional perfusion are the core components for this balance.

Injectable nanocomposite hydrogels broaden this osteoangiogenic concept by integrating H-type vessel induction, copper-containing bioactive glass, MgO nanoparticles, mesoporous bioactive glass nanoparticles, exosome-functionalized hydrogel systems, and GHK-Cu nanofibers into shape-adaptive matrices [31]. In this setting, angiogenesis is not an isolated VEGF-delivery problem but part of a coupled scaffold microenvironment that also includes macrophage phenotype, matrix stiffness, ion release, and bacterial control.

### 5.3. Osteoimmunomodulation

Bone regeneration is immunologically regulated. The early inflammatory phase must clear debris and recruit cells, but persistent M1-dominant inflammation impairs osteogenesis and promotes fibrosis. M2-like macrophage phenotypes support tissue repair through IL-10, TGF-beta, BMP-2, VEGF, and other paracrine mediators.

The bone–implant interface is the few hundred micrometers around an implant where the outcome is binary—direct bone apposition (osseointegration) or fibrous encapsulation (foreign-body response)—and it is decided within days by the protein corona that forms on the surface and by the timing of the macrophage transition from an M1- to an M2-dominant population, which nanoscale roughness, hydrophilicity, and controlled ion release can bias toward repair [52]. Because a scaffold can increase total bone volume while still failing at the interface, bone-to-implant contact, fibrous interface width, and gap-healing distance should be reported alongside bone volume in craniofacial nanomaterial studies.

Two cranial defect studies provide particularly relevant evidence. Aspirin-loaded mesoporous silica/chitosan hydrogels downregulated IL-6, IL-1 beta, TNF-alpha, and iNOS while upregulating IL-10, CD206, and TGF-beta, resulting in enhanced bone formation via TGF-beta/Smad signaling [24]. Gold-loaded mesoporous silica/chitosan scaffolds promoted M2 macrophage markers, increased BMP-2, TGF-beta1, and VEGF secretion, enhanced osteogenic markers, and improved cranial bone regeneration [25]. These findings suggest that nanomaterial therapies may succeed not by overwhelming the defect with osteogenic factors but by reshaping the inflammatory niche into a pro-regenerative environment.

Nanomaterial-integrated injectable hydrogels add further evidence that immune regulation can be engineered at the material level. Mesoporous bioactive glass nanoparticle hydrogels, laponite–polydopamine nanosheet systems, metformin-loaded mesoporous silica, and cyclodextrin-based nanotherapies have been reported to promote M2-like macrophage polarization, ROS control, and macrophage–BMSC crosstalk in craniofacial or cranial defect models [31,53]. This expands osteoimmunomodulation from a drug-release strategy to a design principle for scaffold chemistry, stress relaxation, and ion presentation.

### 5.4. Antimicrobial and Antibiofilm Function

Infection is one of the most clinically important failure modes in craniofacial reconstruction, especially when hardware is placed near the oral cavity, sinuses, traumatic wounds, or previously infected fields. Nanocoatings can inhibit bacterial adhesion, disrupt membranes, generate ROS, release antimicrobial ions, interfere with DNA replication, or physically damage bacterial membranes via nanotopography [30]. Unlike conventional antibiotics, many nanoparticle mechanisms are multi-targeting, which may reduce the likelihood of single-step resistance.

However, antimicrobial nanomaterials are not automatically safe. Silver, copper, and zinc oxide nanoparticles can be cytotoxic to mammalian cells at higher concentrations. Graphene and carbon nanotubes raise concerns regarding inflammation, genotoxicity, and occupational exposure. Photodynamic systems may be impractical for deep implants. A promising direction is controlled no-release or low-release nanotopography that discourages biofilm formation without sustained tissue nanoparticle exposure.

The cytotoxicity and genotoxicity of silver, copper, and zinc oxide are driven primarily by released ions rather than by the particles themselves, amplified by a Trojan-horse effect in which endocytosed particles dissolve rapidly in acidic lysosomes and deliver a perinuclear ion burst: silver depletes glutathione and collapses mitochondrial membrane potential, copper generates hydroxyl radicals through Fenton-type chemistry, and zinc oxide overwhelms intracellular zinc homeostasis, while genotoxicity arises either directly through particle access to DNA and the mitotic spindle or, more commonly at implant-relevant doses, indirectly through oxidative and inflammation-derived DNA damage, all dose-, size-, shape-, and coating-dependent—which explains contradictory results between studies that omit these parameters—and all narrowing the bacteria-versus-host selectivity margin for redox-sensitive osteoblasts and endothelial cells, so a selectivity index against the relevant bone-forming cell type should be reported.

In this context, four ordered research priorities need to be underlined: (a) the preference towards growth-factor-sparing ionic, topographic, or pharmacological strategies over recombinant proteins; (b) the consideration of osteoimmunomodulation as a primary design specification, with macrophage phenotype reported alongside bone volume; (c) design vascularization first for defects exceeding the diffusion-limited volume; and (d) achievement of antimicrobial functioning from the outset, since infection rather than insufficient osteogenesis is the dominant clinical failure mode, with the field standing to gain more from rigorous comparative large-animal evaluation of a few simple constructs than from further proliferation of multifunctional systems tested only in rodent calvarial defects.

## 6. Additive Manufacturing and Bioprinting as Translational Platforms

### 6.1. Why 3D Printing Is Central to Craniofacial Translation

The complex geometry of craniofacial defects makes additive manufacturing more than a fabrication convenience. It enables patient-specific fit, controlled porosity, defect mirroring, preoperative planning, reduced intraoperative shaping, and reproducible scaffold architecture. Clinical translation is most advanced for acellular patient-specific implants using titanium, PEEK, alpha-tricalcium phosphate, hydroxyapatite, and beta-tricalcium phosphate [8,11,20].

The clinically relevant question is no longer whether 3D printing can produce craniofacial implants, but how to add biological activity without making the product unsafe or unapprovable. Acellular nanomaterial-enhanced scaffolds may have a more feasible near-term pathway than cell-laden bioprinted constructs because they avoid advanced therapy medicinal product classification in some jurisdictions.

The development pathway is increasingly better understood as a continuum from electrospinning to melt electrowriting, extrusion printing, bioprinting, and converged biofabrication. Electrospun nanofibers mimic collagen fibrillar architecture and have been widely used for GTR/GBR membranes, while melt electrowriting provides finer architectural control and can be combined with extrusion printing to create multiphasic bone–periodontal ligament interfaces [54]. Craniofacial 3D-printed scaffold reviews identify pore size, material selection, drug loading, and patient-specific CT-to-CAD workflows as core design variables rather than post hoc manufacturing details [13,23].

### 6.2. Bioprinting and Cell-Laden Constructs

Bioprinting allows spatial deposition of cells, hydrogels, bioinks, growth factors, and nanoparticles. Systematic review evidence across oral and craniomaxillofacial tissues identifies periodontal, dental-pulp, bone, temporomandibular joint cartilage, oral mucosa, and salivary gland applications [9]. In bone reconstruction, bioinks may include gelatin, collagen, alginate, hyaluronic acid, decellularized extracellular matrix, PEG, nanohydroxyapatite, graphene oxide, PLGA nanospheres, and multi-material composites.

Cell-laden bioprinting is conceptually attractive because it can recapitulate living tissue architecture, but it remains translationally difficult. Challenges include cell viability during printing, vascularization, the mechanical weakness of hydrogels, bioink reproducibility, sterility, GMP manufacturing, patient-specific cell sourcing, ethical concerns, and regulatory classification as a cell-device combination or advanced therapy product. For cranial vault reconstruction, acellular nanomaterial scaffolds with host cell recruitment may therefore be more clinically realistic in the near term.

Current craniofacial bioprinting reviews classify extrusion, laser-assisted, stereolithography, magnetic, spheroid, and AI-assisted approaches, with bioinks including collagen, gelatin, alginate, hyaluronic acid, GelMA, PEGDA, beta-TCP, bioactive glass, platelet-rich plasma, and nanosilicate-containing NICE-type formulations [12]. The most translationally relevant near-term bioprinted platforms may be acellular or minimally manipulated hydrogel–ceramic constructs that can be standardized, sterilized, and implanted rapidly, whereas autologous cell-laden constructs will require stronger GMP workflows and regulatory justification.

For acellular craniofacial scaffolds, printability, degradation kinetics, and mechanical stability should clearly be prioritized considering polymer choice. PCL offers excellent printability, long-term shape retention, and slow degradation rates, whereas PLGA/PLLA-based polymers are associated with more clinically suitable resorption profiles, and may still generate local acidic degradation pro-inflammatory products. Hence, polymer–ceramic composites are preferable, particularly PCL–nanohydroxyapatite, PLGA/PLLA–β-tricalcium phosphate, and hydroxyapatite–PLLA/PGA, as the ceramic phase improves the osteoconductivity, stiffness, radiopacity, and buffering of acidic degradation products. Ceramic content should nonetheless be optimized with a view to avoiding impaired printability and excessive brittleness [21,22,32,33].

### 6.3. Design Parameters: Pores, Caps, Degradation, and Mechanics

Pore geometry is not a secondary detail. Pores must be large and interconnected enough for vascular and cellular ingrowth, yet scaffolds must retain mechanical integrity. Evidence from beta-TCP/dipyridamole platforms suggests that a pore size around 500 micrometers and solid cap designs can improve calvarial bone formation by guiding ingrowth and excluding soft tissue competition [11]. In VEGF-PLGA/TCP scaffolds, pore sizes around 500 micrometers supported cell adhesion and endothelial responses in vitro [29]. These observations align with broader craniofacial scaffold principles: porosity, interconnectivity, degradation rate, and stiffness must be matched to the anatomical site.

Degradation is equally important. A scaffold that persists too long may act as a chronic foreign body; one that resorbs too fast may collapse before new bone forms. Site-specific resorption is relevant, as calcium phosphate scaffold degradation can differ between calvarial and alveolar sites [11]. Nanomaterials that release ions or drugs must be designed so that biological activity aligns temporally with the inflammatory, reparative, and remodeling phases of bone healing.

The expanded evidence base supports more explicit design ranges. Classical craniofacial biomaterial reviews cite approximately 200–350 micrometers as an osteogenic pore target and 150–500 micrometers for vascularization, while cranial-specific 3D-printed scaffold reviews suggest that larger 500–1000 micrometer pores may be advantageous for cranial vascularization and fusion [13,15,55]. Recent nanomaterial scaffold reviews also emphasize site-specific degradation, with 3–6 months often proposed for craniomaxillofacial applications, whereas slower degradation may be acceptable or necessary in other skeletal sites [15,56]. These ranges should not be interpreted rigidly; the optimal architecture depends on material stiffness, pore interconnectivity, cap design, fixation, defect site, and whether the construct is load-bearing.

Whether excessively large pores impair cell adhesion deserves a direct answer, because the relationship is not monotonic. Below approximately 100 micrometers, vascular ingrowth is restricted and the scaffold center becomes hypoxic, so this represents a firm lower bound [55]. Above approximately 800 to 1000 micrometers, three effects converge to reduce performance. The adhesion surface available per unit scaffold volume falls, so fewer cells attach per construct. Cells that cannot bridge the strut spacing remain rounded rather than spread, with fewer focal adhesions and reduced cytoskeletal tension, and consequently with reduced RUNX2 and osteogenic marker expression, since osteogenic commitment of mesenchymal stromal cells is spreading- and tension-dependent. Tissue deposition is also curvature-driven and proceeds fastest on concave surfaces of small radius, so very large pores fill disproportionately slowly [57,58,59,60]. Compressive strength, in addition, falls steeply with increasing porosity, which matters wherever the construct is subject to loading. Our recommended range is therefore 300 to 600 micrometers for most craniomaxillomandibulofacial applications, with approximately 500 micrometers as a defensible default supported by the beta-TCP/dipyridamole and VEGF-PLGA/TCP data cited above [11,29], extending toward 800 micrometers only where cranial vascularization rather than mechanical support is the limiting factor [13]. Total porosity should exceed 50 per cent, interconnection windows should measure at least 100 micrometers, and strut geometry, material stiffness, and cap design should be reported alongside nominal pore size, because a 500 micrometer pore in a stiff ceramic and the same pore in a soft hydrogel do not present the same environment to a cell.

## 7. Translational Evidence by Anatomical Indication

### 7.1. Cranial Vault and Calvarial Defects

The cranial vault has the strongest preclinical nanomaterial evidence among the anatomical regions reviewed. Rat calvarial models are widely used for proof-of-concept testing of hydrogels, mesoporous silica systems, iron oxide hydrogels, magnesium phosphate bioinks, and immunomodulatory scaffolds [24,25,26,27]. These models are useful for screening but have limitations: they are small, relatively non-load-bearing, heal differently from human cranial defects, and do not replicate the scale or clinical complexity of craniectomy, tumor resection, or pediatric vault reconstruction (Table 3).

The most advanced cranial vault translational platform is 3D-printed beta-TCP coated with dipyridamole, validated across rabbit, sheep, and minipig models, including pediatric craniofacial contexts [11]. The combination of patient-specific CAD/CAM design, optimized pore geometry, pharmacological osteogenic enhancement, and large-animal validation makes this a leading near-clinical strategy.

Cranial-vault-specific systematic review evidence further supports the need for dedicated cranial design criteria rather than extrapolation from long-bone models. Khorasani and Vahidi highlight intramembranous ossification, patient-specific skull curvature, cranial defect geometry, pore architecture, FEA/CFD optimization, and the limitations of rodent and rabbit models as central determinants of translation [13]. Direct calvarial studies among the references include PLA/G-nHA/PRP scaffolds with 83.7% defect fill at 12 weeks [32], PLA/HTGO–simvastatin scaffolds with near-complete rat calvarial repair at 8 weeks [33], and nanosilicate-reinforced cell-laden gelatin–alginate bioprints with significantly higher BV/TV at 12 weeks than controls [40]. Broader nanomaterial scaffold reviews also document earlier cranial or skull evidence for nanostructured PLLA, carbon nanotubes, and TiO2 nanoscaffolds, including pig skull fusion data [49].

### 7.2. Mandibular Defects

Mandibular defects demand higher mechanical performance and infection control than many cranial vault defects. The u-HA/PLLA/PGA sheet study in rat mandibular angle defects provides relevant early evidence for bioresorbable osteoconductive materials [21]. However, future translation requires large-animal mandibular load-bearing models, comparison with titanium fixation or vascularized bone flaps, and biomechanical testing under occlusal loading.

For segmental mandibular defects, nanomaterial scaffolds alone are unlikely to replace vascularized autologous bone in the near term. More realistic near-term roles include adjunctive coatings, guided bone regeneration membranes, reconstruction plate surface modifications, bioactive spacers, dental implant site augmentation, and local delivery systems that improve osseointegration or reduce infection.

The new mandibular and alveolar evidence strengthens this adjunctive translation pathway. Beta-TCP/PDLLA and beta-TCP/PLLA/PGA nanobiomaterials provide rat mandibular critical-size defect data with favorable handling, pore ingrowth, early osteogenic marker expression, and periosteal activity [34,35]. Functional scaffold reviews also describe canine mandibular GBR membranes, nHA/polyamide composites in rabbit mandibular defects, CPC/gelatin cement in canine saddle-type defects, and gelatin/beta-TCP/BMP-2 sponges in rat mandibular defects, underscoring that maxillofacial translation is likely to proceed first through membranes, packing materials, and local delivery scaffolds rather than full segmental replacement [16]. Dental and oral regenerative medicine reviews provide clinical comparator context for GBR, rhPDGF-BB, FGF-2, and patient-specific additive-manufactured oral/craniofacial constructs [61].

### 7.3. Maxillary, Alveolar, and Dentoalveolar Defects

The maxilla is habitually grouped with the mandible, yet the two present opposite reconstructive problems. The maxilla is a thin-walled, largely non-load-bearing buttress structure separating the oral cavity from the nasal cavity, the maxillary sinus, and the orbit, so its dominant requirements are compartment separation, volume stability for dental rehabilitation, and tolerance of an environment in which the graft is frequently exposed to sinus mucosa and to oral flora across a mucosal suture line. Graft incorporation is favored by its cancellous-dominant architecture and rich regional blood supply. Nevertheless, the thin cortical shell offers little primary stability for fixation, while defects arising after maxillectomy, cleft alveolar repair, or advanced periodontal loss appear to be more contained rather than segmental. These features render the maxilla and the dentoalveolar complex the most amenable to particulate, injectable, and membrane-based nanomaterial strategies, while maintaining them as the least dependent on load-bearing performance. The clinical comparators are correspondingly different from those of the mandible. Deproteinized bovine bone mineral, autologous particulate grafts, and guided bone regeneration membranes define the standard of care for alveolar ridge preservation and sinus augmentation, and any nanomaterial proposed for these indications must be tested against them rather than against an empty defect [1,61].

Nanohydroxyapatite-containing composites, calcium phosphate cements, and PLGA-based local delivery systems are the most plausible candidates, and functional scaffold reviews describe nanohydroxyapatite/polyamide composites, calcium phosphate cement/gelatin systems, and growth-factor-loaded sponges in relevant animal models [16]. The evidence required for this region is therefore not further critical-size defect work but randomized comparison against deproteinized bovine bone mineral or autografts, with dental implant survival, volumetric ridge stability at twelve months, and histomorphometric bone-to-implant contact as endpoints, since these are the outcomes on which clinical adoption in this region will actually be judged. For precisely this reason, maxillary and dentoalveolar applications arguably represent the shortest route to a first genuinely comparative clinical trial of a nanomaterial in this anatomical territory.

### 7.4. Orbital, Midfacial, and Esthetic Contour Defects

Evidence for orbital, zygomatic, nasal, and midfacial nanomaterial reconstruction remains sparse. Clinical hydroxyapatite patient-specific implant cases include orbital rim and mandibular angle applications, suggesting potential for non-load-bearing contour reconstruction [20]. Thin, curved orbital anatomy may benefit substantially from patient-specific hydroxyapatite or nanostructured bioceramic implants, provided brittleness, fixation, and long-term stability are addressed.

This remains a major evidence gap for nanomaterial-specific bone regeneration, but the bioprinting and regenerative medicine literature provides further clinical context. Najafi et al. summarize zygomatic and orbital clinical applications of 3D-printed bioceramic and titanium systems, including BGS-7 bioceramic zygomatic cases, patient-specific electron-beam-melted titanium implants, orbital floor comparisons showing improved volume restoration, and office-based fabrication workflows [12]. Latimer et al. similarly describe CAD/CAM-guided pre-bent orbital implants with reduced theater time and improved anatomical fit [61]. Future studies should test nanostructured hydroxyapatite, calcium phosphate, or titanium-hydroxyapatite hybrid implants in orbital and midfacial models with outcomes relevant to contour accuracy, ocular motility, sinus exposure, infection, fixation, and imaging artifacts.

### 7.5. Pediatric Reconstruction

Pediatric craniomaxillofacial reconstruction introduces unique requirements: growth accommodation, suture patency, avoidance of donor-site morbidity, and long-term remodeling. Dipyridamole-coated 3D-printed beta-TCP scaffolds are notable because they have been evaluated in skeletally immature animal models with attention to craniofacial symmetry and suture patency [11]. The companion craniofacial scaffold review emphasizes that dipyridamole-enhanced beta-TCP may preserve suture patency, whereas rhBMP-2 can risk premature suture fusion in craniofacial models [23]. Foundational craniofacial biomaterials literature also recommends resorption profiles compatible with growth in pediatric craniofacial use [15]. This provides a stronger translational rationale than many adult-only rodent studies.

Nanomaterials for pediatric use must meet a higher safety bar because implanted materials interact with developing skeletal and immune systems over decades. Long-term biodegradation, systemic nanoparticle exposure, cranial growth effects, and imaging follow-up are essential endpoints.

## 8. Safety, Nanotoxicology, and Regulatory Translation

### 8.1. Nanoscale-Specific Safety: Cytotoxicity, Genotoxicity, and Nanotoxicology

Before entering this part of the discussion, a terminological clarification is initially required. Nanotoxicology and cytotoxicity are not identical terms. The first denotes the study of hazards arising specifically from nanoscale properties: the very high surface-area-to-volume ratio that increases reactivity and ion dissolution; the formation of a protein corona that confers a biological identity independent of bulk composition; cellular uptake through endocytic routes unavailable to bulk or micron-scale material; the biopersistence of non-degradable particles; and translocation across biological barriers with subsequent distribution to organs remote from the implant site. Cytotoxicity, by contrast, is a measurable endpoint, namely loss of cell viability, which may be produced by nanoscale and non-nanoscale materials alike and which, in the case of dissolving nanoparticles, is frequently attributable to released ions rather than to the nanostructure itself. In this review, cytotoxicity, genotoxicity, and biocompatibility are therefore used as endpoint terms; nanotoxicology is reserved for the discipline and for those hazards that would not arise from the same material in bulk form; and nanoparticle biodistribution and translocation are named explicitly wherever they are meant. Rather than being merely semantic, this distinction determines expectations by regulatory evidence: an ionic cytotoxicity problem is addressed by release-rate control, whereas a nanoscale-specific hazard requires particle-level characterization together with biodistribution and clearance data.

Nanotoxicology is not an optional discussion; it is central to translation. Nanoparticles can change biological behavior according to size, shape, charge, surface chemistry, protein corona formation, aggregation, dose, degradation, and release kinetics. Potential hazards include cytotoxicity, genotoxicity, oxidative stress, complement activation, immune dysregulation, thrombogenicity, chronic inflammation, liver or spleen accumulation, local tissue toxicity, occupational exposure during drilling or machining, and unpredictable behavior after sterilization [4,6,30,48].

Metallic nanoparticles require special scrutiny. Gold nanoparticles may accumulate because they are not readily biodegradable. Silver nanoparticles can accumulate in organs and have narrow therapeutic windows. Copper and zinc oxide nanoparticles can be mammalian-cell toxic at higher concentrations. Iron oxide nanoparticles may be safer in some contexts and offer imaging advantages, but long-term iron metabolism and local accumulation must be characterized. Carbon nanotubes and graphene derivatives raise particular regulatory concerns related to pulmonary and genotoxic effects. Craniofacial nanomaterial reviews also highlight potential central nervous system access through olfactory pathways, oxidative stress, inflammatory reactions, dose dependency, and incomplete knowledge of metabolic clearance, which are especially relevant near the nasal cavity, skull base, and cranial vault [14].

Particle size and degradation behavior should be reported explicitly. A recent nanomaterial scaffold review proposes 10–100 nm as a practical safety window for many scaffold-associated nanoparticles, while particles larger than 100 nm may increase embolic or aggregation risks and particles smaller than 10 nm may be rapidly cleared or distributed systemically [56]. These ranges are material- and route-dependent rather than universal, but they illustrate the type of quantitative safety justification needed for regulatory review.

### 8.2. Manufacturing and Sterilization

Manufacturing reproducibility is a major barrier for nanomaterials. Regulators will require control over particle size distribution, morphology, surface area, crystallinity, charge, aggregation state, purity, endotoxin, residual solvents, degradation products, drug loading, and release kinetics. For 3D-printed implants, layer resolution, porosity, pore interconnectivity, mechanical strength, sterilization compatibility, and batch-to-batch reproducibility must also be validated.

Sterilization deserves specific emphasis. Heat, gamma irradiation, ethylene oxide, plasma, or autoclaving can alter polymer degradation, nanoparticle dispersion, drug activity, surface chemistry, brittleness, and mechanical performance. Reviews of additive manufacturing for bone regeneration highlight that sterilization of printed constructs is under-studied relative to raw materials [8].

### 8.3. Regulatory Classification

Regulatory classification depends on jurisdiction, composition, internal exposure, primary mode of action, drug or biologic inclusion, and whether cells are present. Nanomaterial-containing medical devices may face high-risk classification, particularly under EU MDR Rule 19 when internal exposure to nanomaterials is medium or high [48]. Cell-laden constructs may be classified as advanced therapy medicinal products or combination products, substantially increasing evidence requirements [9].

The most feasible near-term translational pathways may therefore involve acellular, patient-specific, mineral-based scaffolds; coatings using materials with an existing clinical precedent; drug repurposing strategies such as dipyridamole; and PLGA-based release systems with well-characterized components. Historical precedents exist: nanostructured calcium phosphate cement was approved by the United States FDA for craniofacial defect repair in 1996, showing that regulatory translation is achievable for certain mineral nanoscaffolds [49]. At the same time, bioactive 3D-printed cranial scaffolds may be treated as high-risk devices requiring substantial premarket evidence, and cell-laden bioprinted constructs add combination-product or advanced therapy complexity [9,12,13,48]. Novel multifunctional nanomaterials may ultimately be more powerful, but they face a longer regulatory path.

## 9. Proposed Translational Framework

Table 1 summarizes the major nanomaterial classes and their translational roles. Taken together, the evidence supports a tiered interpretation of translational readiness. The first tier comprises near-clinical platforms, particularly 3D-printed calcium phosphate or hydroxyapatite patient-specific implants and beta-TCP/dipyridamole scaffolds. These systems are supported by a combination of anatomical suitability, patient-specific manufacturing workflows, established mineral chemistry, early human experience, or large-animal craniofacial validation. Their next translational step is not further mechanistic proof, but prospective clinical evaluation, long-term imaging follow-up, and comparison with current reconstructive standards.

The second tier comprises platforms that are translationally promising but remain preclinical. This includes mesoporous silica immunomodulatory systems, magnesium and nanosilicate bioinks, iron oxide hydrogels, VEGF- or PLGA-based delivery systems, and injectable nanocomposites. These technologies provide strong biological rationale and encouraging rodent or rabbit data, but they require larger-animal validation, long-term biodistribution and degradation studies, sterilization testing, dose optimization, and clinically relevant comparator groups before they can be considered for human translation.

The third tier comprises speculative or high-risk technologies, including graphene derivatives, carbon nanotubes, cell-laden bioprinting, extracellular-vesicle or lipid-nanoparticle systems, and complex multi-drug or multi-ion constructs. These platforms may ultimately provide advanced biological control, but their current translational pathway is limited by their uncertain long-term fate, manufacturing complexity, regulatory classification, safety concerns, and the lack of clinically relevant craniofacial evidence. For these systems, the immediate priority should be rigorous safety characterization and simplified construct design rather than premature movement toward clinical application.

## 10. Future Directions

### 10.1. Move Beyond Rodent Calvarial Models

Rodent calvarial defects are useful screening models but insufficient for translational claims. Future studies should use staged evidence: in vitro mechanism, rodent proof-of-concept, rabbit or sheep cranial defect, minipig cranial or mandibular model, and finally prospective clinical pilot trials. Large-animal models should include the clinically relevant defect size, fixation method, soft tissue coverage, vascularity, infection risk, and imaging follow-up.

### 10.2. Integrate Antimicrobial Function into Bone-Regenerative Scaffolds

Most bone regeneration scaffolds focus on osteogenesis and vascularization but ignore infection. Future constructs should incorporate antibiofilm design from inception. Particularly promising strategies include copper-containing coatings that combine angiogenesis and antimicrobial activity, nanostructured titanium surfaces with low release, and local antibiotic or antiseptic delivery through mesoporous carriers. These must be tested in contaminated craniofacial defect models, not only sterile calvarial models.

### 10.3. Prioritize Growth-Factor-Sparing Platforms

Recombinant growth factors are powerful but costly and complication-prone at high doses. Ionic signaling, dipyridamole, magnesium phosphate, bioactive glass, iron oxide, and immune-modulating nanoparticles offer alternative routes. The most clinically viable platforms may be those that use familiar materials and pharmacological agents rather than entirely novel biologic-device constructs.

### 10.4. Develop Regulatory-Ready Characterization Standards

Every nanomaterial scaffold should report a minimum translational data package: particle size and distribution, zeta potential, morphology, crystallinity, surface chemistry, endotoxin, sterilization effects, degradation kinetics, release profile, protein corona behavior, local and systemic biodistribution, cytotoxicity, genotoxicity, immunotoxicity, hemocompatibility where relevant, mechanical fatigue, and imaging compatibility. Without these data, promising laboratory results will remain non-translatable.

### 10.5. Use AI-Guided and Patient-Specific Design Responsibly

Machine learning can optimize pore geometry, release profiles, scaffold stiffness, and patient-specific fit. However, algorithmic design must remain interpretable and compatible with regulatory documentation. For medical devices, AI optimization should not replace biological validation; it should guide hypothesis generation and design refinement.

Cranial scaffold reviews now describe FEA for stress distribution, CFD for nutrient and fluid transport, machine learning for biomechanical prediction, and digital twins as future tools for cranial defect reconstruction [13]. Bioprinting reviews also identify AI/ML roles in CT segmentation, bioink viscosity optimization, and real-time print monitoring, although clinical AI-integrated craniofacial bioprinting evidence remains absent [12]. These tools should be treated as design-control aids and not as substitutes for animal, mechanical, sterility, and clinical performance testing.

## 11. Limitations of This Review

This manuscript is based on a curated evidence set rather than a formal systematic review with database search strings, duplicate screening, risk-of-bias assessment, and a PRISMA flow diagram. The available literature is heterogeneous, spanning reviews, regulatory documents, in vitro studies, rodent models, large-animal preclinical work, and small clinical case evidence. Direct comparison across platforms is limited by differences in defect size, animal species, scaffold geometry, endpoints, follow-up duration, and reporting standards. Many cited nanomaterial systems are promising but remain far from clinical validation. The evidence-quality concern is not theoretical: a systematic review of cranial scaffolds reported the frequent lack of randomization and blinding in animal studies, as well as a moderate risk of bias in non-randomized evidence [13].

The review also highlights a publication imbalance: calvarial and mandibular models are well represented, whereas orbital, zygomatic, nasal, and midfacial reconstruction are under-studied despite emerging clinical additive-manufacturing case evidence [12,61]. Infection and antimicrobial function remain insufficiently integrated into bone-regenerative nanomaterial studies. Finally, long-term nanotoxicology and biodistribution data are absent for many platforms, preventing firm clinical recommendations. Clinical translation can also fail despite biologically plausible design; one retrieved 3D-printed PCL periodontal scaffold showed dehiscence at 14 months with granulomatous tissue and islands of new bone, illustrating the gap between scaffold concept and durable human success [54].

## 12. Conclusions

Nanomaterial-based therapies offer a compelling path toward biologically active, patient-specific reconstruction of craniomaxillofacial and cranial vault defects. The field is moving from passive scaffold replacement toward multifunctional constructs that combine osteoconduction, osteoinduction, angiogenesis, immunomodulation, antimicrobial protection, controlled release, and anatomical precision. The strongest translational signals currently come from 3D-printed calcium phosphate and hydroxyapatite-based patient-specific scaffolds, especially those supported by large-animal cranial data or early human case experience. Immunomodulatory mesoporous silica systems, iron oxide hydrogels, magnesium phosphate bioinks, VEGF-releasing PLGA constructs, and antimicrobial nanocoatings provide powerful mechanistic advances but remain largely preclinical.

The decisive next step is not simply to discover more nanomaterials, but to validate rationally designed platforms through anatomy-specific models, long-term safety testing, manufacturable processes, sterilization-compatible workflows, and clear regulatory pathways. For clinical craniomaxillofacial surgery, the most realistic near-term future is likely to involve acellular 3D-printed scaffolds or patient-specific implants enhanced with controlled nanomaterial bioactivity, rather than fully cell-laden bioprinted constructs. If these systems can demonstrate safety, reproducibility, and superiority over existing standards, nanomaterial-based reconstruction may progress from bench innovation to routine bone restoration.

Mapped by anatomical region rather than by material class, the picture remains uneven, but still in an informative way. The strongest evidence and the clearest near-term route are emphasized by the cranial vault, as its defects are non-load-bearing, geometrically well defined, and already reconstructed routinely with patient-specific implants. The shortest path to a genuinely comparative clinical trial is, however, offered by the maxilla, given that validated comparators and accepted endpoints already exist for ridge preservation and sinus augmentation. The mandible remains the hardest problem, and nanomaterials will most plausibly enter mandibular reconstruction as coatings, membranes, and local delivery systems attached to conventional load-bearing solutions rather than as replacements for vascularized bone. The orbit and midface are under-studied relative to their clinical volume, and pediatric reconstruction, where resorbable growth-compatible constructs would be of greatest value, has the least long-term safety data of all.

The safety and regulatory bottom line is that the principal deficit of this field is no longer mechanistic insight but characterization discipline. Released ion concentration over time, particle-size distribution, biodistribution and clearance, sterilization-induced changes in degradation and release, and batch-to-batch reproducibility are reported inconsistently or not at all in most of the studies reviewed here, and it is their absence, rather than any specific toxicological finding, that will delay approval. Constructs that remain acellular, that use materials with an existing clinical precedent, and that avoid recombinant biologics will move through the device pathway substantially faster than those that do not, and this consideration should shape design at the bench rather than being confronted at the point of submission.

On this basis, five priorities arise. First, comparative large-animal studies in anatomically appropriate models should replace further rodent calvarial screening for platforms that have already demonstrated proof of principle. Second, growth-factor-sparing ionic and pharmacological strategies should be advanced ahead of recombinant protein delivery. Third, antimicrobial function should be integrated into bone-regenerative design from the outset rather than added as an afterthought. Fourth, a minimum reporting package covering particle characterization, release kinetics, degradation, biodistribution, and sterilization stability should become an expectation for publication in this field. Finally, the maxilla and the mandible should be studied and reported separately, since evidence generated in one does not transfer to the other. Clinical arrival, when it comes, will most likely be signaled not by a novel multifunctional construct but by a simple, manufacturable, sterilizable, acellular, patient-specific scaffold that outperforms titanium or autografts on infection, revision, and contour retention in a controlled trial. Producing that evidence, rather than producing further materials, is the work that remains.

## Figures and Tables

**Table 1:** Functional nanomaterial classes for craniomaxillofacial and cranial vault reconstruction.

Nanomaterial Class	Main Function	Representative Platform	Most Relevant	Translational	Key Limitation	Relevant
			Indication	Maturity		References
Nano-hydroxyapatite	Osteoconduction,	3D-printed HA PSI; u-	Non-load-	Early clinical	Brittleness,	[20,21,22]
and calcium phosphate	mineral mimicry	HA/PLLA/PGA sheet	bearing CMF	to preclinical	limited load-	
			contour;		bearing strength	
			mandibular			
			adjuncts			
Beta-tricalcium	Osteoconductive	3D-printed beta-TCP with	Cranial vault,	Advanced	No definitive	[11,23]
phosphate	resorbable scaffold	dipyridamole	alveolar,	preclinical	human trials	
			pediatric CMF			
Mesoporous silica	Drug delivery, Si-	ASA/MSN/chitosan; Au-	Cranial critical-	Rodent	Biodistribution	[24,25]
nanoparticles	mediated	MSN/chitosan	size defects	preclinical	and long-term	
	osteogenesis				clearance	
Iron oxide	Mechanical	Fe_2_O_3_/GelMA hydrogel	Craniofacial	Rodent	Long-term iron	[26]
nanoparticles	reinforcement,		bone defects	preclinical	accumulation	
	angiogenesis,				unknown	
	imaging					
Magnesium phosphate	Growth-factor-	ECM/AMP bioink	Cranial vault	Rodent	Incomplete	[27]
/Mg-based systems	independent		bioprinting	preclinical	defect closure;	
	osteogenesis				scale-up	
VEGF/PLGA and	Controlled	VEGF-PLGA/TCP scaffold	Large	In vitro to	Biologic release	[28,29]
polymeric carriers	angiogenic delivery		vascularization- limited defects	preclinical	and combination- product	
					complexity	
Silver/copper/zinc	Antimicrobial and	Ti or PEEK nanocoatings	Infection-prone	In vitro to	Cytotoxicity and	[30]
oxide nanocoatings	antibiofilm function		implants	limited	release control	
				clinical		
				precedent		
Nanotopographic	Osseointegration	Anodized TiO_2_ nanotubes,	Titanium CMF	Preclinical to	Manufacturing	[10,30]
surfaces	and anti-adhesion	nanopits	hardware	device	reproducibility	
				translation		
Injectable	Shape-adaptive	NM-integrated	Irregular	Rodent/rabbit	In situ gelation	[31]
nanocomposite	osteogenesis,	GelMA/alginate/MBGN/laponite	craniofacial	preclinical	control and	
hydrogels	immunomodulation,	systems	cavities,		biodistribution	
	angiogenesis		calvarial			
			adjuncts			
PLA/PCL–calcium	Printable	PLA/G-nHA/PRP, PLA/HTGO–	Calvarial,	Rodent/rabbit	Slow polymer	[22,32,33]
phosphate	osteoconductive	simvastatin, PCL-nHAp	alveolar,	preclinical	degradation and	
nanocomposites	scaffold and drug		contour		fixation	
	delivery		adjuncts			

**Table 2:** Translational readiness of representative platforms.

Evidence Level	Platform Examples	Current Evidence	Translation Gap	Relevant References
In vitro proof of mechanism	VEGF-PLGA/TCP scaffold	Endothelial proliferation, ALP increase, release kinetics	In vivo vascularized bone formation	[29]
Rodent cranial or mandibular validation	ASA/MSN/CSH, Au-MSN/chitosan, Fe_2_O_3_/GelMA, ECM/AMP, u-HA/PLLA/PGA, beta-TCP/PDLLA, beta-TCP/PLLA/PGA, PLA/G-nHA/PRP, PLA/HTGO–simvastatin, nSi/Gel-Alg bioprints	Improved bone volume, osteogenic markers, immunomodulation, calvarial or mandibular defect fill	Large-animal validation and long-term safety	[21,24,25,26,27,32,33,34,35,40]
Large-animal translational package	3D-printed beta-TCP/dipyridamole	Rabbit, sheep, minipig cranial data; pediatric model	Human clinical trial	[11,23]
Early human clinical evidence	3D-printed HA patient-specific implants	Small case series, short follow-up, radiological osteogenesis	Larger cohorts, long-term outcomes, comparative trials	[20]
Regulatory/clinical adoption	Nano-HA dental/orthopedic products; silver wound dressings	Approved devices in related fields	CMF-specific indications and outcomes	[10,30,48]

**Table 3:** Proposed indication-specific nanomaterial strategy.

Defect Type	Dominant Clinical Requirement	Preferred Near-Term Platform	Add-On Nanotechnology	Required Next Evidence	Relevant References
Cranial vault non-load-bearing defect	Contour, protection, osseointegration	3D-printed calcium phosphate or HA PSI	Dipyridamole, Mg ions, Fe oxide imaging	Large-animal and human comparative trials	[11,13,20,23,26]
Pediatric cranial reconstruction	Growth compatibility, suture preservation	Resorbable beta-TCP scaffold	DIPY coating; controlled degradation	Long-term-growth in animal models	[11,15,23]
Mandibular segmental defect	Load-bearing strength, infection control	Titanium or vascularized graft adjunct	HA nanocoating, antimicrobial nanocoating	Load-bearing large-animal studies	[16,21,34,35]
Alveolar ridge or sinus augmentation	Osteoconduction and volume stability	Calcium phosphate or xenograft composite	nHA, PLGA growth-factor carriers	RCTs against DBBM/autograft	[1,16,61]
Infected or contaminated CMF field	Biofilm prevention	Titanium/PEEK scaffold or plate	Cu/Ag/ZnO or no-release nanotopography	In vivo infected defect models	[14,30]
Orbital or midfacial contour	Shape accuracy, low artifact, biocompatibility	HA or hybrid PSI	Nanostructured surface for osseointegration	Orbital/midface clinical series	[12,20]

## Data Availability

No new data were created or analyzed in this study. Data sharing is not applicable to this article.

## Abbreviations

ALP	Alkaline phosphatase
AM	Additive manufacturing
AMP	Amorphous magnesium phosphate
ATMP	Advanced therapy medicinal product
BMP	Bone morphogenetic protein
BMSC	Bone marrow mesenchymal stromal cell
CAD/CAM	Computer-aided design/computer-aided manufacturing
CMF	Craniomaxillofacial
CT	Computed tomography
DBBM	Deproteinized bovine bone mineral
DIPY	Dipyridamole
DPSC	Dental pulp stem cell
ECM	Extracellular matrix
EMA	European Medicines Agency
FDA	United States Food and Drug Administration
GBR	Guided bone regeneration
GelMA	Methacrylated gelatin
GMP	Good manufacturing practice
HA	Hydroxyapatite; hyaluronic acid, depending on context
HIF	Hypoxia-inducible factor
MD	Medical device
MDR	Medical Device Regulation
MSN	Mesoporous silica nanoparticle
NP	Nanoparticle
PEEK	Polyetheretherketone
PLGA	Poly(lactic-co-glycolic acid)
PSI	Patient-specific implant
ROS	Reactive oxygen species
TCP	Tricalcium phosphate
VEGF	Vascular endothelial growth factor
